# Supplementary material for: Comparing the timeliness and adequacy of antenatal care uptake between women who married as child brides and adult brides in 20 sub-Saharan African countries
Source: PLoS One. 2022 Jan 13;17(1):e0262688. doi: 10.1371/journal.pone.0262688 (PMC8758032; doi:10.1371/journal.pone.0262688)
Supplement: Attachment — Submitted filename: Response to Reviewers.docx [file pone.0262688.s002.docx]

Response to Editor’s and reviewer’s comments
==============================

ACADEMIC EDITOR:

**Comments**

Dear authors on your scholarly work; you have brought an important study problem in the area of practice. However, the manuscript has some language usage flaws including punctuations, wordings, spelling and grammar errors. These problems are found throughout the manuscript. Moreover, there are some minor methodological limitations as the reviewer raised. Therefore, please make repeated proof-reading and thorough copyediting before resubmitting the manuscript. This would help increase the readability of the manuscript if published.

**Response**

Thank you for the comments. We have done a thorough editing of the manuscript and addressed the problems on punctuations, spelling and grammar errors. We have also addressed the minor methodological issues raised.

**Reviewers' comments:**

Reviewer #2: The authors have done a good job in addressing the previous round of reviewer comments and the manuscript reads well now. Thank you for your detailed responses to my comments, much appreciated!

**Response**

Thank you.

**Comments**

A few minor comments:
1. In the response to reviewer comments, you mentioned that data on age at first pregnancy is not available in the DHS and age at first birth was available for very few countries. I would add this point to your methods section too. You have added the point about why you used age at first marriage from a theoretical perspective but this will also clarify why you had to do this from a practical perspective.

**Response**

As advised, we have included the suggested information in the methods section of the manuscript as shown below:

*We could not use variables such as age at first pregnancy and age at first birth because the former is not available in the DHS while the latter is only available for a few countries.*

**Comments**

2. Your conclusion states that from a policy perspective, ending early marriage is the solution to improving ANC use. Agreed, these two concepts are related, but perhaps also consider the idea that improving girls/women's socio-economic wellbeing, which - as acknowledged clearly in your discussion now - is a driver of both ANC use and age at marriage, should perhaps be the key focus of policy makers/programmes?

**Response**

Thank you for the suggestion. We have included the additional recommendation in the manuscript as shown below:

*Besides, considering the precarious conditions of child brides, including low education, poor employment prospects and generally low socio-economic status, interventions to improve the situations of girl-children must be a major focus and consideration of policymakers.*

**Comments**

3. Table 2 - typo in heading: 'parentage' instead of 'percentage'

**Response**

We have changed ‘parentage’ to ‘percentage’
